# A full scale comparative study of methods for generation of functional Dendritic cells for use as cancer vaccines

**DOI:** 10.1186/1471-2407-7-119

**Published:** 2007-07-03

**Authors:** Silvija Jarnjak-Jankovic, Hege Hammerstad, Stein Sæbøe-Larssen, Gunnar Kvalheim, Gustav Gaudernack

**Affiliations:** 1 Department of Pediatric Research, The National Hospital, Oslo, Norway; 2 Section for Immunotherapy, The Norwegian Radium Hospital, University of Oslo, Norway; 3 Department of Cellular Therapy, The Norwegian Radium Hospital, University of Oslo, Oslo, Norway

## Abstract

**Background:**

Dendritic cells (DCs) are professional antigen-presenting cells with the ability to induce primary T-cell responses and are commonly produced by culturing monocytes in the presence of IL-4 and GM-CSF for 5–7 days (Standard DC). Recently, Dauer and co-workers presented a modified protocol for differentiation of human monocytes into mature DCs within 48 hours (Fast DC). Here we report a functional comparison of the two strategies for generation of DCs from human monocytes with adaptions for large-scale clinical use.

**Methods:**

The Elutra Cell Selection System was used to isolate monocytes after collection of leukapheresis product. The enriched monocytes were cultured in gas permeable Teflon bags with IL-4 and GM-CSF for 24 hours (Fast DC) or 5 days (Standard DC) to obtain immature DCs. The cells were then transfected with mRNA from the leukemia cell line Jurkat E6 by electroporation and incubated for additional 24 h or 2 days in the presence of pro-inflammatory cytokines (TNFα, IL-1β, IL-6 and PGE_2_) to obtain mature DCs.

**Results:**

Mature Fast DC and Standard DC displayed comparable levels of many markers expressed on DC, including HLA-DR, CD83, CD86, CD208 and CCR7. However, compared to Standard DC, mature Fast DC was CD14^high ^CD209^low^. Fast DC and Standard DC transfected with Jurkat E6-cell mRNA were equally able to elicit T cell specifically recognizing transfected DCs in vitro. IFNγ-secreting T cells were observed in both the CD4+ and CD8+ subsets.

**Conclusion:**

Our results indicate that mature Fast DC are functional antigen presenting cells (APCs) capable of inducing primary T-cell responses, and suggest that these cells may be valuable for generation of anti-tumor vaccines.

## Background

Dendritic cells (DCs) are potent antigen-presenting cells (APCs) involved in the induction of T-cell-mediated immune responses and have appeared as important candidates for cellular-based therapies [1]. Immature DCs are found throughout the body where they capture and process antigens (Ag). When activated by danger signals the DCs start differentiation towards a mature penotype and migration to the T cell dependent areas of secondary lymphoid organs. During this process, they lose the capacity for Ag-capturing and upregulate major histocompatibility complex (MHC)- and costimulatory molecules for stimulation of naive T cells [2-4].

In vitro, DCs can be differentiated from various cellular sources, including CD34+ progenitor cells from bone marrow (BM) and cord blood (CB), and monocytes obtained from peripheral blood mononuclear cells (PBMCs) [5]. For clinical DC vaccines, monocytes have been the general source for DC generation [6]. Monocyte-derived DCs can be generated from PBMCs collected by leukapheresis. Different monocyte enrichment techniques are available including plastic adherence, immunomagnetic separation as well as elutriation. By using the Elutra^® ^Cell Separation System (Gambro BCT), up to 20 × 10^9 ^PBMCs can be elutriated within one hour, which makes this device convenient for large scale isolation of monocytes for further generation of DCs for clinical purposes. Immature DCs can be generated from monocytes by culturing for 5 days in serum-free medium containing IL-4 and GM-CSF [7,8], and further matured using pro-inflammatory cytokines as described previously [9].

Dauer and co-workers have developed a protocol for the generation of DCs from human monocytes within 48 hours (Fast DC) [10,11]. Novel culture systems aimed to reduce processing time for generation of DCs are likely to become important for large-scale clinical production of DCs. The aim of the present study was to investigate if this protocol could be adapted for large-scale clinical use involving collection of PBMCs by leukapheresis, isolation of monocytes by using the Elutra™ Cell Separation System, and culturing in sterile Teflon bags. Using such a set-up, we have compared DCs generated by the Fast DC and standard protocols with respect to transfection efficiency, DC phenotype, and T cell stimulatory capacity following transfection with whole tumor mRNA.

## Methods

### Collection of peripheral blood mononuclear cells

PBMCs were collected by leukapheresis from patients with advanced prostate cancer included in clinical trials of DC vaccines [12] using Cobe Spectra (Gambro BCT). The experiments were approved by the Regional Committee for Medical Research Ethics and performed in compliance with the World Medical Association Declaration of Helsinki. Written informed consent was obtained from all patients.

### Isolation and culture of enriched monocytes in sterile VueLifeTM FEP Teflon bags

Monocytes were purified from PBMCs using the Elutra™ Cell Separation System (Gambro BCT). This is a semi-automatic, centrifuge-based method using continuous counter-flow elutriation technology to separate cells into multiple fractions based on size and density. This procedure resulted in a recovery of 75–90% of all monocytes with a purity of 65–77% of CD14 positive cells. Between 0.9–1.3 × 10^9 ^monocytes were isolated and cultured at a final concentration of 10^6^cells/ml in Teflon bags with serum-free CellGro DC medium supplemented with 2500 U/ml of GM-CSF and 1000 U/ml of IL-4 (CellGenix). The cells were collected after 24 hours (Fast DC) or 5 days (Standard DC) and transfected with mRNA isolated from the leukemia cell line Jurkat E6 as described previously [9]. After removal of samples for flowcytometry and contamination test, the cells were incubated for additional 24 hours for FastDCs and 48 hours for Standard DC in CellGro DC medium supplemented with a maturation cocktail of proinflammatory cytokines:10 ng/ml IL-1α; 1000 U/ml IL-6; 10 ng/ml TNF-α (CellGenix) and 10 ng/ml PGE2 (Sigma Aldrich). A fraction of the cells was cryopreserved as described previously [13].

### Cell lines

The leukemia cell line Jurkat E6 was obtained from the American Type Culture Collection (ATCC). The cell line was cultured in RPMI 1640 medium, supplemented with penicillin (100 U/mL), streptomycin (100 μg/mL) and 10% fetal calf serum (FCS) (all from Sigma Aldrich). Cells were maintained at 37°C in a humidified atmosphere supplemented with 5% CO_2_.

### Preparation of mRNA from Jurkat E6 cells

Total RNA was isolated from 25 × 10^6 ^Jurkat E6 cells using Trizol Reagent (Invitrogen, Basel, Switzerland). Poly (A) + mRNA was isolated from total RNA using the GenoPrep Direct mRNA kit (GenoVision, Oslo, Norway). mRNA was either used fresh or stored at -70°C until use.

### Transfection of immature DCs with mRNA

Immature DCs were collected from the Teflon bags, washed twice, and resuspended in 0.6–0.8 ml cold culture DC medium and placed in a 4°C cooling block. mRNA transfection was performed by electroporation as described earlier [9]. To measure transfection efficiency, cells were electroporated with EGFP-pCIpA102 mRNA [14] encoding the enhanced green fluorescence protein and analysed by flow cytometry (FACSCalibur, Becton Dickinson). Cells electroporated without mRNA (mock DCs) were used as negative control.

### Flowcytometry measurements

Immature and mature DCs were phenotyped using a four-color panel of monoclonal antibodies: CD14 APC, Anti-HLA-DR PerCp, CD86 FITC (all BD Bioscience, San Jose, CA), CD1a FITC (DAKO A/S, Denmark), CD83 PE, CD209 PE, CD208 PE (Immunotech, France) and CCR7 APC (R&D Systems). Isotype-matched antibodies were used for negative controls. Cells were analyzed using the FACSCalibur flow cytometer and CellQuest software (BD Biosciences).

### Test for in vitro stability of mature phenotype (Washout test)

Fully mature DCs are capable of retaining their phenotype following culture in DC medium without any cytokines. Frozen mature DCs were thawed and cultured in DC medium alone. After 24 and 48 hours in culture the DCs phenotype was analyzed using Flow Cytometry. The quality control of the frozen-produced DCs consisted of sterility tests, phenotyping and viability testing by trypan blue staining before freezing and after thawing.

### Generation of T-cell responses in vitro

Thawed autologous PBMCs were plated in 6-well plates, 20 × 10^6 ^cells in 3 ml of DC medium per well, and incubated for 2 hours at 37°C in 5% CO2. Non-adherent T lymphocytes were collected and used as responder cells: 3 × 10^6 ^cells in 1 ml of DC medium supplemented with antibiotic. Thawed mRNA-transfected DCs used as stimulator cells were washed once and irradiated with 300 Gy. After washing, the cells were resuspended in DC medium at a final concentration of 0.3 × 10^6 ^cells/ml. To each well containing responder cells, 1 ml of the prepared DCs was added. The Negative Isolation Kit from (Dynal, Biotech) was used for isolation of CD4 and CD8 T cells according to the manufacturer's protocol. Isolation was performed on day 7 after in vitro priming and ELISPOT assay was performed essentially as described earlier [9]. Briefly, 7 day primed T cells (50–400.000 cells per well) were tested with 5000 mock transfected control DCs or the same number of transfected DCs in plates coated with anti IFNgamma antibodies overnight in duplicate cultures. Spot forming cells were counted manually the next day.

## Results

### Morphological comparison of Fast DC with Standard DC

**Figure 1:**
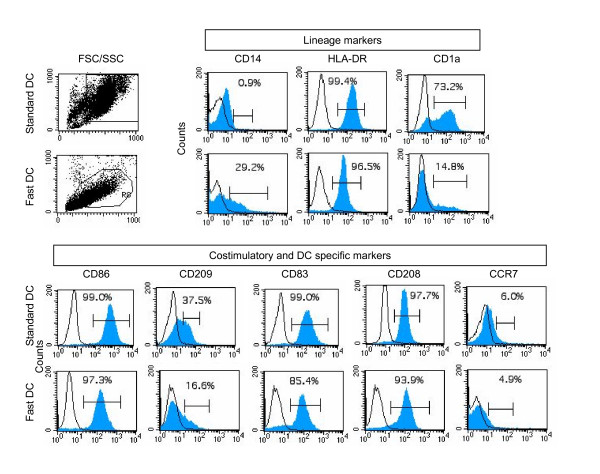
Comparative phenotypic analysis of Standard DC and mature FastDCs by flow cytometry. Surface expression of a panel of antigens was determined following maturation with TNFα, IL1β, IL6 and PgE2 for 24 hours (mature FastDCs) and 48 hours (Standard DC). Open diagram shows reactivity with control mAbs. Closed diagrams shows reactivity with anti HLA-DR and CD antibodies. The setting of the gates based on the forward (FSC) and site scatter (SSC) is indicated to the left in the figure. One representative of three performed experiments is presented.

The morphology of Fast DC was compared to Standard DC by fluorescent microscopy of EGFP transfected DCs. Fast DC in the mature stage were similar to monocytes in size, but they developed cytoplasmic protrusions. In contrast, Standard DCs were larger in size and had long motile cytoplasmic processes ('veils') typical of terminally differentiated dendritic cells. Flow cytometric analysis (Fig. 1) confirmed the fluorescent microscopy findings.

### Immunophenotype of Fast and Standard DC

Phenotypic characteristics of Fast DC have previously been reported by Dauer et al [10] for small scale laboratory production. In three parallel experiments, 200 × 10^6 ^monocytes were cultured for DC development and an average of 98 × 10^6 ^immature DCs were obtained after 24 hours of incubation, resulting in a mean of 53 × 10^6 ^mature DCs after additional 24 hours in culture (26,5% of initial monocytes seeded). The cell recovery and viability of mature Fast DC and Standard DC were similar (results not shown) [5]. The phenotypic analysis of mature Fast DC showed that about 93–98% of cells had high levels of HLA-DR. After 24 hours of maturation, Fast D showed up regulation of CD83 and CD86 (Fig. 1). Fast DC expressed significantly lower amounts of CD1a and CD209 than Standard DC. About 30% of the Fast DC expressed the monocyte marker CD14, indicating that 48 hrs was not sufficient for complete down-regulation of this monocyte marker.

### Stability of immunophenotype

**Figure 2:**
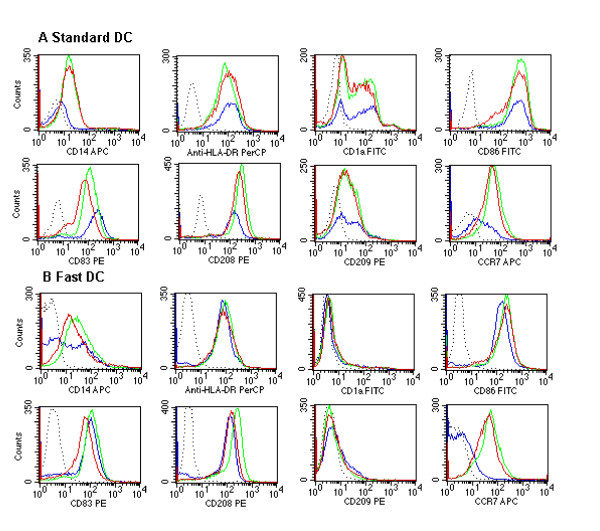
Phenotypical stability of mature dendritic cells, Standard DC (a) and Fast DC (b), after freezing/thawing and incubation without cytokines. Dotted line, isotype-matched control; blue line, before freezing/thawing; green line, 24 hrs in culture without cytokines; red line, 48 hrs in culture without cytokines. One of three performed experiments is presented.

To investigate if the Fast DC had gained a stable phenotype during the short differentiation period the cells were frozen and thawed and incubated without exogenously added cytokines for 48 hrs. The results show that a stable phenotype of the DCs was obtained for both cell types (Fig 2a–b). One exception was CD14, which seemed to some extent to re-appear on Standard DC and not diminish further on Fast DC. Interestingly, incubation of Fast DC under cytokine-free conditions led to increased expression of CCR7 over time (Fig. 2b), similar to the levels seen in Standard DC.

### Efficiency of mRNA transfection in different types of DCs

**Figure 3:**
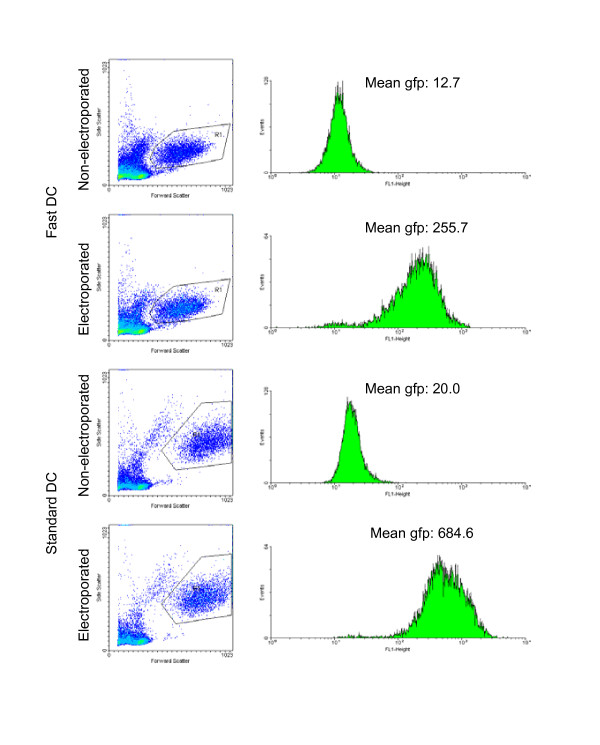
mRNA transfection of DCs. Flow cytometric analysis of mature FastDCs and Standard DC after transfection with EGFP/pCIpA_102 _mRNA (10 μg/400 μl) by square-wave electroporation and maturation for 24 hours in medium with maturation cocktail. Control cells were incubated on ice with EGFP mRNA without electrophoresis, washed and incubated for 24 hrs. Conditions for electroporation were 500 volt and 1-ms when using a 4-mm-gap cuvette.

Electroporation with EGFP mRNA was performed after 5 days in culture for Standard DC, and after 24 hours for Fast DC. The electroporation was performed at 500 volt for 1 millisecond in a 4-mm cuvette using the BTX ECM-830 square-wave electroporator (Genetronics Inc., San Diego, CA). The viability of transfected Fast DC and Standard DC was similar to non-transfected cells, and the transfection rate was close to 100% (Fig. 3). The average green fluorescence in EGFP-mRNA transfected DCs was 2.7 times higher in Standard DC compared to mature Fast DC, probably reflecting the difference in size (Fig. 3).

### T-cell stimulatory capacity of FastDCs and Standard DC

**Figure 4:**
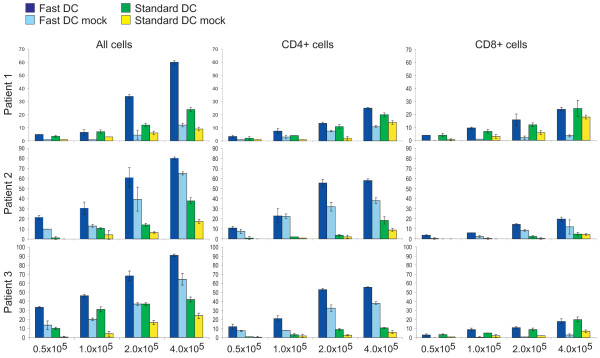
ELISPOT analysis of T-cell activation. T cells were stimulated with DCs transfected with tumor mRNA or control DCs (mock transfected) as indicated. Mean number of IFN γ positive spots from duplicate wells obtained with the indicated numbers of un-fractionated T cells, and CD4+ and CD8+ T-cell subsets from individual patients. Results are presented as mean +/- standard deviation (SD).

Tumour mRNA-loaded FastDCs and Standard DC were co-cultured with autologous T cells in serum free DC medium, without addition of cytokines. After 7 days, IFN-γ production from autologous T cells co-cultured with mRNA transfected Fast DC or Standard DC were analyzed in an ELISPOT assay (Fig. 4). T cells primed for one week with thawed antigen-loaded DCs demonstrated a specific T-cell response against mRNA-loaded DCs compared to mock DCs. This was observed for both Fast DCs and Standard DC. The total number of spot-forming cells was proportional to the number of T cells added to the wells. This was also seen with mock transfected DCs. Priming with Fast DC in general produced more IFN-γ-secreting T cells compared to Standard DC, both with mRNA- and mock-transfected DCs, resulting in a higher background. When considering the differences in the number of IFN-γ-secreting T cells between mRNA- and mock-transfected DCs, Fast DC and Standard DC appeared to be equally efficient in producing specific T-cell responses against the mRNA encoded antigens. To address which of the T-cell subpopulations were responsible for the IFN-γ secretion in the ELISPOT assay, CD4+ and CD8+ T cells were isolated using negative selection with Dynabeads coated with CD8 and CD4 antibodies respectively and analysed in separate assays. The results (Fig. 4) demonstrate that both CD4+ and CD8+ responses were obtained in all patients. While Standard DC activated comparable numbers of CD4+ and CD8+ T cells, the T cell response against Fast DC seemed to be dominated by CD4+ T cells.

## Discussion

Our experiments demonstrate that mature Fast DC can be generated from monocytes within 48 hours in a large-scale clinical setting. Furthermore our results indicate that priming of a T cell response by Fast DC and Standard DC following tumour mRNA loading is equally efficient, indicating that Fast DC may substitute for conventional DCs in clinical trials. Some differences between mature Fast DC and conventional monocyte derived DCs were noted. Compared to Standard DCs, Fast DC displayed a mixed phenotype (CD83^+^, CD208^+^, CD86^+^) with some expression of CD14 retained. After analyzing the phenotype of DC maintained in the washout cultures for up to 48 h we observed CD14 re-appear on Standard DC and not diminish further on Fast DC. This results confirmed previous findings, indicating that monocyte-derived DCs stimulated with proinflammatory cytokines may not be terminally differentiated cells [15]. A significant difference between the results reported here and those reported in [10] is the level of CD14 remaining on immature and mature Fast DC. Whereas Dauer et al [10] used positive selection of monocytes by the MACS CD14 isolation kit to obtain monocytes, we used elutriation. Moreover, the two methods also differed in the use of media, sera, GM-CSF concentration and type of culture vessel. We believe that the difference may result from the use of magnetic particles conjugated with CD14 antibodies in the Dauer protocol. The fact that CD14 is bound by the antibodies on the particles and thus internalized by the monocytes during subsequent culture may explain the complete loss of CD14 during the in vitro culture with DC differentiating cytokines in their protocol. CD1a was significantly lower expressed in Fast DC than in Standard DC, while other markers such as CD83, CD208 and CCR7 were expressed on a similar proportion of both cell types. The lower expression of CD1a is probably not of any functional importance in the context of mRNA loaded DC to be used as cancer vaccines, since this molecule functions to present bacterial lipids to T cells. Expression of CD209 was low in mature Fast DC and was lost almost completely upon incubation of the cells in the absence of cytokines. CD209 or DC-SIGN (Dendritic cell-specific ICAM-3 grabbing nonintegrin) plays important roles in macrophage and DC recognition of pathogens but seem not to be required for T cell activation [16]. CD209 thus probably exerts its main role in antigen uptake and processing, rather than in the phase were antigen is presented to the T cells. In mature Fast DC the expression of CCR7 increased over time (after 24 hours and 48 hours), to some extent this was also the case in the Standard DC. The chemokine receptor CCR7 is a key molecule for the entry of lymphocytes and dendritic cells into secondary lymphoid organs and their homing to T cell and B cell zones. Although Fast DC were morphologically smaller and with less surface membrane protrusions, overall, the data on the phenotype of mature Fast DC indicates that these cells compare favorably with conventional DCs and supports the suggestion that such cells may be ready for clinical trials.

In our approach to clinical studies [12,17], the ability of DCs transfected with mRNA isolated from tumor cell line or tissue to induce T cell responses is important. Immature Fast DC was readily transfected with mRNA encoding EGFP. More importantly, when immature Fast DC were transfected with mRNA isolated from Jurkat E6 leukemia cell line, which was used as a tumor model, T cells specific for transfected DCs were readily picked up in the IFNγ ELISPOT assay after one single cycle of in vitro stimulation of autologous T cells. Both CD4+ and CD8 + T cells were evident. Due to a prominent autologous mixed leukocyte reaction (MLR) seen when DCs are co-incubated with autologous T cells, background reactivity is often a problem in the immuno monitoring assays. The same phenomenon is observed in patients vaccinated with DCs and auto-MLR reactive T cells from DC-vaccinated patients can be characterized at the clonal level. [18-20]. This seems also to be the case when mature Fast DC are used.

Production of Standard DC under GMP conditions for clinical use is time, cost and labor intensive and represents a bottleneck in personalized vaccine development. Dauer et al [10] recently reported that mature Fast DC could be obtained following the same conditions as for Standard DC with phenotypic characteristics as well as functional properties similar to DCs generated by the standard protocol. We have adapted the method of Dauer to full-scale GMP production of Fast DC required for clinical trials. Compared with common standard 7-days protocol, this new strategy simplified the process and reduced labor, cost and time for the whole experimental procedure. In the clinical setting this may provide an opportunity to treat more patients in a shorter period of time. Based on previous pre-clinical experiments [9,17,5], we are currently engaged in clinical trials using mRNA transfected Standard DC as cancer vaccines [12].

## Conclusion

The present experiments using full-scale production demonstrate that adequate numbers of mature and functional Fast DC for clinical vaccines can be obtained in a reproducible manner from patients with advanced cancer and paves the way for implementing this method in our clinical trials.

## Competing interests

The author(s) declare that they have no competing interests.

## Authors' contributions

SJJ carried out the immunological studies, participated in the flow cytometry analysis and drafted

the manuscript.

HH carried out the flow cytometry analysis.

SSL carried out the mRNA transfection with EGFP and analysis of data.

GK participated in its design and coordination of the study.

GG conceived of the study, and participated in its design and coordination.

## Pre-publication history

The pre-publication history for this paper can be accessed here:



## References

[B1] Celluzzi CM, Welbon C (2003). Dendritic cell culture: a simple closed culture system using ficoll, monocytes, and a table-top centrifuge. Journal of hematotherapy & stem cell research.

[B2] Cella M, Engering A, Pinet V, Pieters J, Lanzavecchia A (1997). Inflammatory stimuli induce accumulation of MHC class II complexes on dendritic cells. Nature.

[B3] Austyn JM (1992). Antigen uptake and presentation by dendritic leukocytes. Seminars in immunology.

[B4] Schnurr M, Then F, Galambos P, Scholz C, Siegmund B, Endres S, Eigler A (2000). Extracellular ATP and TNF-alpha synergize in the activation and maturation of human dendritic cells. J Immunol.

[B5] Mu LJ, Lazarova P, Gaudernack G, Saeboe-Larssen S, Kvalheim G (2004). Development of a clinical grade procedure for generation of mRNA transfected dendritic cells from purified frozen CD34(+) blood progenitor cells. International journal of immunopathology and pharmacology.

[B6] Romani N, Gruner S, Brang D, Kampgen E, Lenz A, Trockenbacher B, Konwalinka G, Fritsch PO, Steinman RM, Schuler G (1994). Proliferating dendritic cell progenitors in human blood. The Journal of experimental medicine.

[B7] Sallusto F, Lanzavecchia A (1994). Efficient presentation of soluble antigen by cultured human dendritic cells is maintained by granulocyte/macrophage colony-stimulating factor plus interleukin 4 and downregulated by tumor necrosis factor alpha. The Journal of experimental medicine.

[B8] Curti A, Ferri E, Pandolfi S, Isidori A, Lemoli RM (2004). Dendritic cell differentiation. J Immunol.

[B9] Jarnjak-Jankovic S, Pettersen RD, Saeboe-Larssen S, Wesenberg F, Olafsen MR, Gaudernack G (2005). Preclinical evaluation of autologous dendritic cells transfected with mRNA or loaded with apoptotic cells for immunotherapy of high-risk neuroblastoma. Cancer gene therapy.

[B10] Dauer M, Obermaier B, Herten J, Haerle C, Pohl K, Rothenfusser S, Schnurr M, Endres S, Eigler A (2003). Mature dendritic cells derived from human monocytes within 48 hours: a novel strategy for dendritic cell differentiation from blood precursors. J Immunol.

[B11] Obermaier B, Dauer M, Herten J, Schad K, Endres S, Eigler A (2003). Development of a new protocol for 2-day generation of mature dendritic cells from human monocytes. Biol Proced Online.

[B12] Mu LJ, Kyte JA, Kvalheim G, Aamdal S, Dueland S, Hauser M, Hammerstad H, Waehre H, Raabe N, Gaudernack G (2005). Immunotherapy with allotumour mRNA-transfected dendritic cells in androgen-resistant prostate cancer patients. British journal of cancer.

[B13] Mu LJ, Gaudernack G, Saeboe-Larssen S, Hammerstad H, Tierens A, Kvalheim G (2003). A protocol for generation of clinical grade mRNA-transfected monocyte-derived dendritic cells for cancer vaccines. Scandinavian journal of immunology.

[B14] Saeboe-Larssen S, Fossberg E, Gaudernack G (2002). mRNA-based electrotransfection of human dendritic cells and induction of cytotoxic T lymphocyte responses against the telomerase catalytic subunit (hTERT). Journal of immunological methods.

[B15] Dauer M, Schad K, Junkmann J, Bauer C, Herten J, Kiefl R, Schnurr M, Endres S, Eigler A (2006). IFN-alpha promotes definitive maturation of dendritic cells generated by short-term culture of monocytes with GM-CSF and IL-4. Journal of leukocyte biology.

[B16] Granelli-Piperno A, Pritsker A, Pack M, Shimeliovich I, Arrighi JF, Park CG, Trumpfheller C, Piguet V, Moran TM, Steinman RM (2005). Dendritic cell-specific intercellular adhesion molecule 3-grabbing nonintegrin/CD209 is abundant on macrophages in the normal human lymph node and is not required for dendritic cell stimulation of the mixed leukocyte reaction. J Immunol.

[B17] Kyte JA, Kvalheim G, Aamdal S, Saeboe-Larssen S, Gaudernack G (2005). Preclinical full-scale evaluation of dendritic cells transfected with autologous tumor-mRNA for melanoma vaccination. Cancer gene therapy.

[B18] Kyte JA, Gaudernack G (2006). Immuno-gene therapy of cancer with tumour-mRNA transfected dendritic cells. Cancer Immunol Immunother.

[B19] Kyte JA, Kvalheim G, Lislerud K, Thor Straten P, Dueland S, Aamdal S, Gaudernack G (2007). T cell responses in melanoma patients after vaccination with tumor-mRNA transfected dendritic cells. Cancer Immunol Immunother.

[B20] Kyte JA, Mu L, Aamdal S, Kvalheim G, Dueland S, Hauser M, Gullestad HP, Ryder T, Lislerud K, Hammerstad H, Gaudernack G (2006). Phase I/II trial of melanoma therapy with dendritic cells transfected with autologous tumor-mRNA. Cancer gene therapy.

